# A Casein Kinase II Phosphorylation Site in AtYY1 Affects Its Activity, Stability, and Function in the ABA Response

**DOI:** 10.3389/fpls.2017.00323

**Published:** 2017-03-13

**Authors:** Xiu-Yun Wu, Tian Li

**Affiliations:** ^1^Laboratory of Plant Molecular Biology, Centre for Plant Biology, School of Life Sciences, Tsinghua UniversityBeijing, China; ^2^Institute of Crop Science, Chinese Academy of Agricultural SciencesBeijing, China

**Keywords:** AtYY1, casein kinase II, phosphorylation site, transcriptional activity, protein stability, ABA response

## Abstract

The phosphorylation and dephosphorylation of proteins are crucial in the regulation of protein activity and stability in various signaling pathways. In this study, we identified an ABA repressor, *Arabidopsis* Ying Yang 1 (AtYY1) as a potential target of casein kinase II (CKII). AtYY1 physically interacts with two regulatory subunits of CKII, CKB3, and CKB4. Moreover, AtYY1 can be phosphorylated by CKII *in vitro*, and the S^284^ site is the major CKII phosphorylation site. Further analyses indicated that S^284^ phosphorylation can enhance the transcriptional activity and protein stability of AtYY1 and hence strengthen the effect of AtYY1 as a negative regulator in the ABA response. Our study provides novel insights into the regulatory mechanism of AtYY1 mediated by CKII phosphorylation.

## Introduction

Mammalian Yin Yang 1 (YY1) is an evolutionarily conserved Cys_2_/His_2_ (C_2_H_2_) zinc-finger transcription factor that has a fundamental role in various biological processes including cell growth, cell differentiation, embryonic development and tumorigenesis (Zhang et al., 2011). Recently, a class of YY1 homologs has been identified in many plant species such as maize, *Arabidopsis*, rice and tobacco (Xu et al., 2001; Li et al., 2016). Plant YY1 shows structural similarity with mammalian YY1 protein, which contains four conserved tandem C_2_H_2_ zinc fingers (Xu et al., 2001; Li et al., 2016). Interestingly, plant YY1 also resembles mammalian YY1 in both DNA binding ability and transcriptional activity. For example, *Arabidopsis* YY1 (AtYY1) can bind to a conserved YY1 binding site and has both repression and activation domains (Li et al., 2016). To date, increasing evidence demonstrates that plant YY1 is involved in the regulation of multiple developmental and physiological processes, e.g., hormone responses, photosynthesis and fungal resistance (Xu et al., 2001; Lai et al., 2013; Li et al., 2016). For instance, AtYY1 can act as a repressor in ABA signaling by directly upregulating *ABA REPRESSOR1* (*ABR1*) expression, in addition to negatively regulating ABA-responsive gene expression (Li et al., 2016).

The phosphorylation and dephosphorylation of proteins mediated by kinases and phosphatases plays essential roles in plant response to many stresses and environmental signals and are convenient for the rapid regulation of protein activity and stability in various signaling pathways (Dai et al., 2013). Casein kinase II (CKII) is a ubiquitous serine-threonine protein kinase highly conserved in all eukaryotes and involved in the regulation of essential cellular processes (Ahmed et al., 2002). In *Arabidopsis*, most identified substrates of CKII are light signaling- or circadian clock-related transcription factors, including GBF1 (Klimczak et al., 1995), CCA1 (Sugano et al., 1998), LHY (Sugano et al., 1999), HY5 (Hardtke et al., 2000), and HFR1 (Park et al., 2008). Although existing evidence indicates that CKII is also involved in ABA signaling (Mulekar et al., 2012; Wang et al., 2014), very few of the components in ABA signaling have been identified as CKII targets thus far. In this study, we identified a major CKII phosphorylation site (S^284^) in AtYY1 and found that this site is closely linked to the regulation of AtYY1 functions.

## Materials and Methods

### Yeast Two-Hybrid Assay

The yeast two-hybrid assay was performed as described (Yao et al., 2007). The entire *AtYY1* coding sequence (CDS) was subcloned into the pGBKT7 vector to generate an AtYY1-BD plasmid. The *CKB3* and *CKB4* CDSs were amplified from *Arabidopsis* cDNA and subcloned into the pGADT7 vector to generate the CKB3-AD and CKB4-AD plasmids. Yeast AH109 cells were co-transformed with AtYY1-BD and CKB3-AD or CKB4-AD plasmids. The interaction was determined by the growth of co-transformants on SD/-Trp/-Leu/-His medium and quantitative analysis of β-galactosidase activity using o-nitrophenyl β-D-galactopyranoside (ONPG) as a substrate according to the Yeast Protocols Handbook (Clontech, Palo Alto, CA, USA).

### Bimolecular Fluorescence Complementation (BiFC) Assay

For the BiFC assay, the *AtYY1* CDS was subcloned into the pUC-SPYNE vector to generate an AtYY1-YFP^N^ plasmid, and the *CKB3* and *CKB4* CDSs were subcloned into the pUC-SPYCE vector to generate the CKB3-YFP^C^ and CKB4-YFP^C^ plasmids as described (Walter et al., 2004). The AtYY1-YFP^N^ and CKB3-YFP^C^ or CKB4-YFP^C^ plasmids were simultaneously introduced into onion epidermal cells using the particle bombardment method (Sanford et al., 1993). After incubation in darkness at 22°C overnight, transformed onion epidermal peels were stained with 4′,6-diamidino-2-phenylindole (DAPI) and observed on a Zeiss LSM710 confocal microscope. The wavelengths for YFP and DAPI were 514 and 405 nm for excitation, and 527 and 488 nm for detection, respectively.

### *In vitro* Phosphorylation Assay and Mass Spectrometry Analysis

A point mutation converting S^284^ (AGT) of AtYY1 to A (GCT) or D (GAT) was introduced using a PCR-mediated mutagenesis method (Li et al., 2009). Recombinant His-MBP, His-AtYY1, His-S284D, and His-S284A proteins were prepared as described previously (Li et al., 2016). Human glioblastoma recombinant CKII (α_2_β_2_ tetrameric holoenzyme) was purchased from NEB (New England Biolabs, Ipswich, MA, USA). For the *in vitro* phosphorylation assay (Park et al., 2008), 3 μg of His-MBP, His-AtYY1, His-S284D, or His-S284A protein was incubated with 25 units of CKII, 100 μM ATP, and 1× Protease Inhibitor Cocktail (Sigma, St. Louis, MO, USA) in the CKII reaction buffer (20 mM Tris-HCl, 50 mM KCl, 10 mM MgCl_2_, pH 7.5) in a total volume of 40 μL. The reaction mixture without CKII or recombinant His-tagged proteins was used as the negative control. The reaction was incubated at 30°C for 45 min, then 7 μL of 6× Protein Loading Buffer (TransGen Biotech, Beijing, China) was added, and the sample was boiled for 3 min. Then, the samples were separated with 12% SDS-PAGE, transferred to polyvinylidene fluoride membranes (Millipore Billerica, MA, USA) and immunoblotted with an anti-phosphoserine antibody (Abcam, Cambridge, MA, USA) and an anti-His antibody (Abmart, Shanghai, China), respectively. After washing, blots were incubated with a horseradish peroxidase-conjugated secondary antibody (goat anti-mouse IgG), and then detected using enhanced chemiluminescence reagents (Pierce, Rockford, IL, USA).

After incubation with CKII, the His-AtYY1 protein was subjected to mass spectrometry analysis as reported (Lin et al., 2012). LC-MS/MS analysis was performed on a LTQ-Orbitrap XL mass spectrometer (Thermo, San Jose, CA, USA) coupled online with an Eksgent Nano 2D LC system. The mass spectrometer was operated in data-dependent mode to automatically switch between MS and MS/MS. Survey full scan MS spectra were acquired from m/z 300 to m/z 1800, and the 10 most intense ions with a charge state above 2 and an intensity threshold above 500 were fragmented in the linear ion trap using with normalized collision energy of 35%. The raw data were processed using Proteome Discoverer (version 1.4.0.288, Thermo Fischer Scientific). MS/MS spectra were searched with the SEQUEST engine against the *Arabidopsis* protein database (TAIR release 10, 27416 sequences). Peptide spectral matches (PSM) were validated with a targeted decoy database search at a 1% false discovery rate (FDR). With Proteome Discoverer, peptide identifications were grouped into proteins according to the law of parsimony.

### *In vitro* Degradation Assay

The *in vitro* degradation assay was carried out as previously described (Park et al., 2008). In brief, plant extracts were prepared from 10-day-old wild-type seedlings and resuspended in a cell-free degradation assay buffer (25 mM Tris-HCl, pH 7.5, 10 mM MgCl_2_, 5 mM DTT, 10 mM NaCl, and 10 mM ATP). For degradation of the His-AtYY1, His-S284D and His-S284A recombinant proteins, cell debris was removed by centrifugation before adding to the proteins. The reaction mixtures (total reaction volume 110 μL) containing 300 μg plant extracts and 5 μg recombinant protein were incubated at 25°C, and at different time points (0, 15, 30, 50, or 100 min), 20 μL of the reaction mixture was transferred into new tubes containing 4 μL of 6× Protein Loading Buffer to stop the degradation process. Then, the samples were boiled for 3 min, separated on a 12% SDS-PAGE gel and detected with immunoblot analysis using anti-His antibodies (Abmart, Shanghai, China).

### Assay for Transcriptional Activity in Tobacco Leaves

The reporter and effector plasmids, which included 3 × YY1-LUC-NOS, 3 × mYY1-LUC-NOS, 35S-AtYY1-NOS, 35S-S284A-NOS, and 35S-S284D-NOS were constructed as reported earlier (Li et al., 2016). Transient transformation assays were performed in 4-week-old tobacco leaves using the particle bombardment method (Sanford et al., 1993). Equal quantities (1.0 μg) of reporter plasmids and effector plasmids were used for each bombardment, and the pRTL-NLUC (35S-Renilla LUC-NOS) plasmid was used as an internal control. After bombardment, luciferase (LUC) assays were performed with the Dual-Luciferase Reporter Assay System (Promega, Madison, WI, USA) as described (Li et al., 2016).

### Generation of Transgenic Plants and Phenotypic Analysis

For the *pAtYY1::AtYY1, pAtYY1::S284A* and *pAtYY1::S284D* constructs, a 2.1-kb *AtYY1* promoter was PCR-amplified and subcloned into the pCAMBIA3301 vector. Then, full-length wild-type and mutant *AtYY1.1* (*S284A* and *S284D*) were inserted into the *Bam*HI and *Pml*I sites of the modified pCAMBIA3301 vector under control of its native promoter. Each construct was transfered into *Agrobacterium tumefaciens* strain GV3101 and transformed into *yy1* mutants (SALK_040806C) with the floral dip method (Clough and Bent, 1998).

The seed germination assay and cotyledon greening assays were carried out as described (Li et al., 2016). In brief, approximately 100 seeds from both the wild-type and transgenic plants were planted in triplicate on 0.5× Murashige and Skoog (MS) agar medium with different concentrations of ABA (0, 0.5 or 1.0 μM), and first maintained at 4°C for 2.5 days before being incubated at 22°C in a growth chamber under long-day conditions (16-h light/8-h night). Germination rates were scored 2 days after incubation and cotyledon greening ratios were determined based on the appearance of green cotyledons after 8 days of incubation.

### RNA Extraction and Quantitative Real-Time PCR (qRT-PCR) Analysis

Total RNA was extracted from 10-day-old *Arabidopsis* seedlings treated with 100 μM ABA (0, 3, 6, or 12 h) using TIANGEN RNAplant plus Reagent (Tiangen, Beijing, China). The cDNA was synthesized using the SuperScript II system (Invitrogen, Madison, WI, USA). qRT-PCR was carried out on an Applied Biosystems 7500 Real-time PCR system using the Power SYBR Green PCR MasterMix (Applied Biosystems, Foster City, CA, USA) as previously described (Li et al., 2011). *Actin2* was used as an internal standard. All assays were performed with three biological replicates. Primers used in this study were listed in Supplementary Table S1.

### Statistical Analyses

All data were shown as means ± standard deviation (SD). Statistical analysis was done with a two-tailed, unpaired, Student’s *t*-test. Differences were considered significant at *P* < 0.05.

## Results and Discussion

### The AtYY1 Phosphorylation Site S^284^ is a Predicted CKII Phosphorylation Site

**FIGURE 1:**
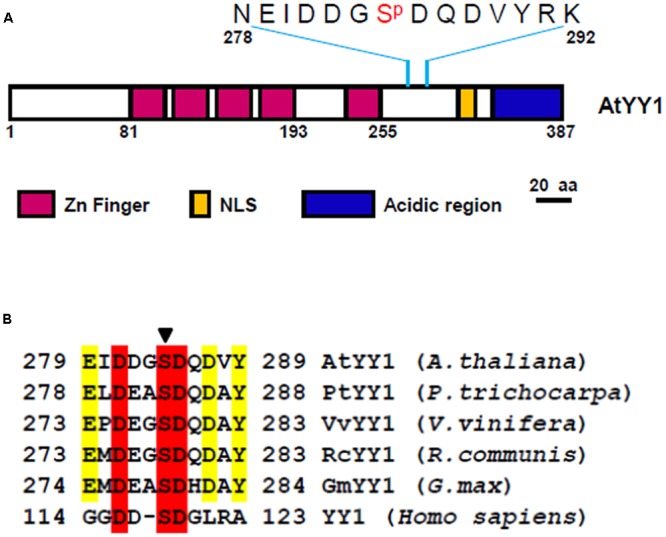
***Arabidopsis* Ying Yang 1 (AtYY1) is a phosphoprotein with an evolutionarily conserved casein kinase II (CKII) phosphorylation site. (A)** Schematic representation of AtYY1 protein and its S^284^ phosphorylation site. Zinc-finger domains (Zn Finger), putative nuclear localization signal (NLS) and the acidic transactivation region are shown as boxes in purple, blue and yellow, respectively. **(B)** The CKII phosphorylation site is evolutionarily conserved among many plant and mammalian YY1 homologs. YY1 (*Homo sapiens*, NP_003394), AtYY1 (*Arabidopsis thaliana*, At4g06634), PtYY1 (*Populus trichocarpa*, XP_002328766), VvYY1 (*Vitis vinifera*, XP_002265428), RcYY1 (*Ricinus communis*, XP_002529014), GmYY1 (*Glycine max*, XP_003525002). The arrow indicates the conserved CKII phosphorylation site.

Recently, a large-scale *Arabidopsis* phosphoproteome study identified AtYY1 as a phosphoprotein with one serine (S) phosphorylation site, S^284^ (Reiland et al., 2009), which is located between its DNA binding domain (zinc finger region) and its acidic transactivation domain (**Figure 1A**). Sequence analysis^1^ revealed that the DDGS^284^DQD sequence of AtYY1 is a predicted casein kinase II (CKII) phosphorylation site and is highly similar to a reported mammalian YY1 CKII phosphorylation site, DDS^118^DG (Riman et al., 2012). Through a bioinformatic approach, we found that predicted CKII phosphorylation sites are present not only in AtYY1 but also in many other plant YY1 homologs (**Figure 1B**), suggesting that YY1 proteins are likely evolutionarily conserved CKII targets.

### AtYY1 Physically Interacts with the CKII Regulatory Subunits CKB3 and CKB4

**FIGURE 2:**
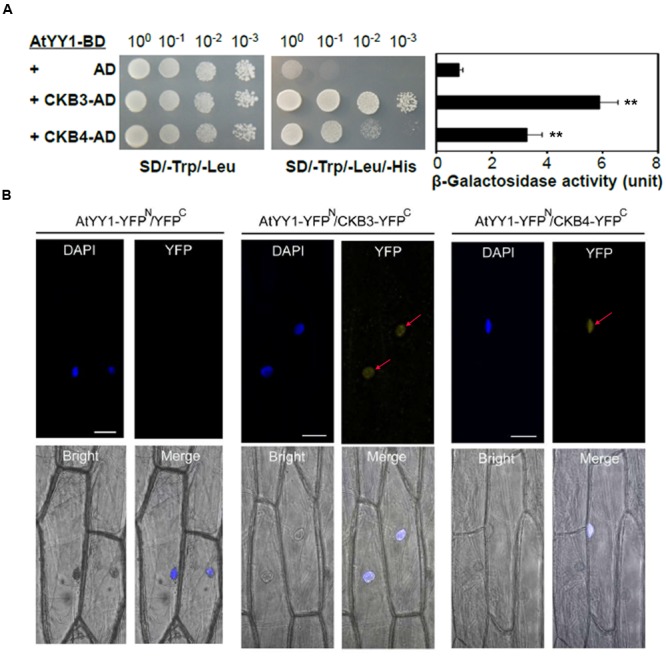
***Arabidopsis* Ying Yang 1 physically interacts with the CKII regulatory subunits CKB3 and CKB4. (A)** AtYY1 interacts with CKB3 and CKB4 in a yeast two-hybrid assay. BD, GAL4 DNA binding domain; AD, GAL4 activation domain. The yeast strains were serially diluted before spotting on selection medium. β-galactosidase activity was assayed for each strain (right panel). ^∗∗^*P* < 0.01 (*t*-test). **(B)** *In vivo* interaction between AtYY1 and CKB3 or CKB4 in the nuclei of onion epidermal cells as determined by BiFC assays. YFP^N^, YFP protein N-terminus; YFP^C^, YFP protein C-terminus. YFP signals were observed in onion cells co-bombarded with the AtYY1-YFP^N^ and CKB3-YFP^C^ or CKB4-YFP^C^ plasmids. No YFP signal was observed in onion cells co-bombarded with the AtYY1-YFP^N^ and YFP^C^ control plasmids. The nuclei were stained with DAPI (4′,6-diamidino-2-phenylindole, blue). Bars = 50 μm.

To confirm the hypothesis that AtYY1 is a target of CKII, we first tested whether AtYY1 can interact with CKII subunits. In *Arabidopsis*, there are four catalytic α-subunits (CKA1–CKA4) and four regulatory β-subunits (CKB1–CKB4) (Salinas et al., 2006). Most of CKII α-subunits and β-subunits are located in the nucleus (Salinas et al., 2006), indicating that these CKII subunits are likely to co-localize and interact with AtYY1 transcription factor *in vivo*. By using a yeast two-hybrid system, we found that AtYY1 interacts moderately with CKB3 and weakly with CKB4, judging from both the yeast growth on a selection plate that lacks histidine and a liquid β-galactosidase assay using ONPG as a substrate (**Figure 2A**). No physical interaction was detected between AtYY1 and other subunits of CKII based on yeast two-hybrid assays.

To further confirm that AtYY1 can interact with CKB3 and CKB4 *in vivo*, we performed bimolecular fluorescence complementation (BiFC) assay in onion epidermal cells. For this, AtYY1 was fused to the N-terminal portion of yellow fluorescent protein (YFP; AtYY1-YFP^N^), and CKB3 and CKB4 were fused to the C-terminal portion of YFP (CKB3-YFP^C^ and CKB4-YFP^C^, respectively). We observed YFP signals in the nuclei of onion cells co-bombarded with the AtYY1-YFP^N^ and CKB3-YFP^C^ or CKB4-YFP^C^ plasmids, while no YFP signal was detected in onion cells co-bombarded with AtYY1-YFP^N^ and YFP^C^ plasmids (**Figure 2B**). These results suggest that AtYY1 interact with CKB3 and CKB4 in the nuclei of plant cells and are consistent with the fact that AtYY1 is a nuclear localized protein (Li et al., 2016). Taken together, these data indicate that AtYY1 can interact directly with CKB3 and CKB4 *in vivo*.

Since *AtYY1* is induced by ABA treatment (Li et al., 2016), we also examined the expression of *CKB3* and *CKB4* under ABA treatment by qRT-PCR. As shown in Supplementary Figure S1, 100 μM ABA treatment (6 h) led to about 1.7-fold and 5-fold increase in *CKB3* and *CKB4* expression, respectively. Similar ABA-inducible expression among *AtYY1, CKB3*, and *CKB4* raised the possibility that their interactions might still occur in the ABA response.

### AtYY1 Can Be Phosphorylated by CKII *In vitro*

**FIGURE 3:**
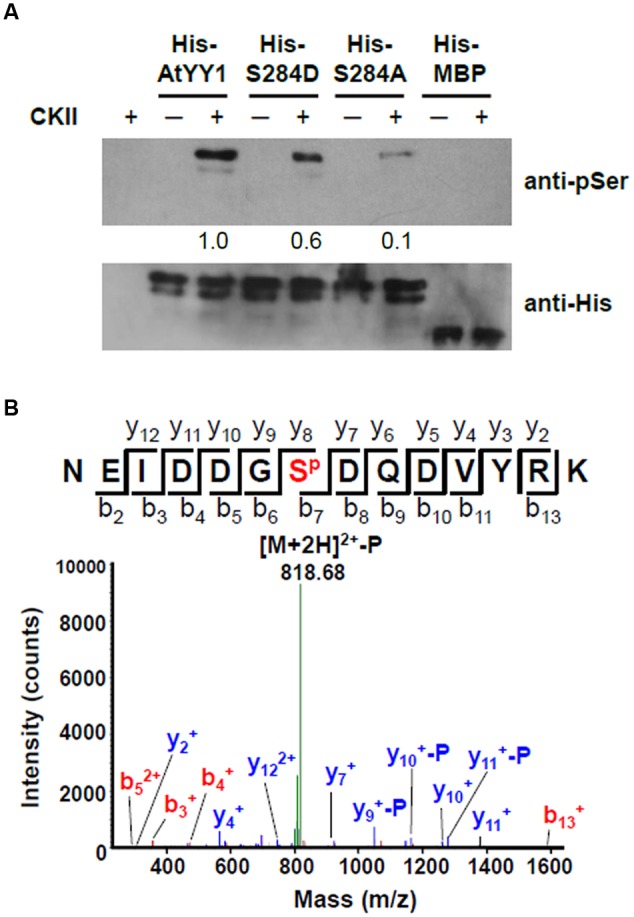
***Arabidopsis* Ying Yang 1 can be phosphorylated by CKII *in vitro* and S^284^ is a major CKII phosphorylation site. (A)** *In vitro* phosphorylation assay of wild-type and mutant AtYY1 proteins by CKII. Phosphorylated proteins were detected by immunoblotting with an anti-phosphoserine antibody. Identical protein loading amounts (3 μg) were confirmed by immunoblotting using an anti-His antibody. Relative phosphorylation signal intensity was labeled below each lane. **(B)** A typical phosphorylated peptide from AtYY1 identified by LC-MS/MS. S^P^ shows that the S^284^ site is phosphorylated. The fragment ions assigned to both y-ions and b-ions are labeled.

Because AtYY1 could interact with two regulatory subunits of CKII, we next tested whether AtYY1 can be phosphorylated by CKII *in vitro*. Recombinant AtYY1 (His-AtYY1) protein and AtYY1 proteins mutated at the S^284^ site (His-S284A and His-S284D) were expressed in *Escherichia coli* and purified as His-tag fusion proteins. These fusion proteins were used as the substrates in an *in vitro* phosphorylation assay and were detected by immunoblotting using an anti-phosphoserine antibody. As shown in **Figure 3A**, His-AtYY1 can be effectively phosphorylated by CKII, whereas His-MBP was not phosphorylated by CKII. Mutating the S^284^ site (His-S284A and His-S284D) clearly decreased the phosphorylation efficiency of CKII, indicating that S^284^ of AtYY1 might be a primary CKII phosphorylation site. Interestingly, the S284A mutation almost abolished the CKII-mediated phosphorylation of AtYY1, whereas the S284D mutation seemed to have less blocking effect than the S284A (**Figure 3A**), possibly because the S284D mutation could mimic the S^284^ phosphorylation in charge and structure, and lead to potential misidentification by the anti-phosphoserine antibody.

The AtYY1 fusion protein was further analyzed by mass spectrometry (MS) after incubation with CKII. Through liquid chromatography tandem MS (LC-MS/MS), most of the identified phosphorylated peptides were phosphorylated by CKII at the S^284^ site (**Figure 3B** and Supplementary Figure S2). It can be noted that other CKII phosphorylation sites (S^225^ and T^266^) were also identified in AtYY1, but their signal intensity was far lower than that of the S^284^ site (Supplementary Figure S2), confirming that the S^284^ site of AtYY1 is a major CKII phosphorylation site.

### The S^284^ Phosphorylation Site Affects the Transcriptional Activity of AtYY1

**FIGURE 4:**
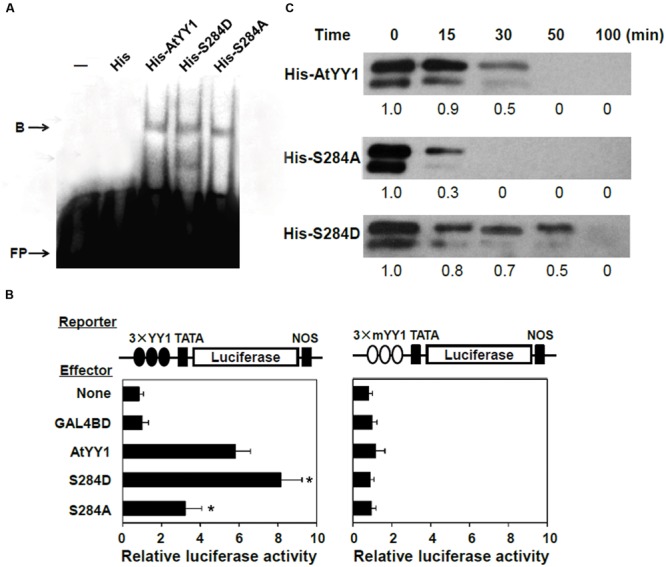
**S^284^ phosphorylation site affects the transcriptional activity and protein stability of AtYY1. (A)** EMSA showing that S^284^ site mutation does not affect the DNA binding ability of AtYY1. Purified His-tagged protein (1 μg) was incubated with biotin-labeled probe (100 fmol) from the *ABR1* promoter. B, shift band; FP, free probe. **(B)** The effect of S^284^ site mutation on AtYY1 transcriptional activity. The LUC reporter was driven by three tandem wild-type or mutant YY1 binding sites (3 × YY1 or 3 × mYY1). ^∗^*P* < 0.05 (*t*-test) indicates the difference from the AtYY1 effector. **(C)** The effect of S^284^ site mutation on AtYY1 protein degradation. Purified wild-type or mutant His-tagged AtYY1 proteins were incubated with plant extracts for different periods of time and were then detected by immunoblotting with an anti-His antibody. Relative amounts of wild-type or mutant AtYY1 proteins were labeled below each lane.

To explore the effect of S^284^ phosphorylation on AtYY1 function, we first examined whether its phosphorylation could affect the DNA binding ability of AtYY1. S284D and S284A of AtYY1 were used to simulate the phosphorylation and dephosphorylation of S^284^, respectively, due to the fact that A or D has high chemical similarity (charge and structure) with dephosphorylated or phosphorylated S (Park et al., 2008; Tellinghuisen et al., 2008). Using an electrophoretic mobility shift assay (EMSA), we found that mutations at the S^284^ site, including S284D and S284A, did not change the DNA binding ability of AtYY1 (**Figure 4A**), possibly because S^284^ is not located in the region of the DNA binding domains (tandem zinc fingers).

Next, the effect of S^284^ phosphorylation on AtYY1 transcriptional activity was investigated using a transient expression assay. We constructed a luciferase (LUC) reporter driven by three tandem 27-bp *ABR1* promoter sequences (–534 ∼ –508) containing the YY1 binding site (3 × YY1-LUC-NOS) or a mutant YY1 site (3 × mYY1-LUC-NOS) as described previously (Li et al., 2016). For the 3 × YY1-LUC-NOS reporter, co-transfection with an effector plasmid that encodes AtYY1 resulted in transactivation of the LUC reporter gene (**Figure 4B**), consistent with the hypothesis that AtYY1 is a positive regulator of *ABR1* (Li et al., 2016). Compared with wild-type AtYY1, S284D had showed increased transactivation of the LUC reporter, whereas S284A decreased the transcriptional activity (**Figure 4B**). In contrast, transcription of the LUC reporter gene driven by the promoter containing mutant YY1 sites was not affected by AtYY1, S284D, or S284A (**Figure 4B**). These results suggest that S^284^ phosphorylation or dephosphorylation may enhance or reduce the transcriptional activity of AtYY1.

### The S^284^ Phosphorylation Site Affects the Degradation Rate of AtYY1 *In vitro*

As reported earlier, phosphorylation of mammalian YY1 by CKII can enhance its protein stability by preventing protease cleavage (Riman et al., 2012). Thus, we carried out experiments to test whether the S^284^ phosphorylation site can affect the stability of AtYY1, similar to its homologs in mammals. Recombinant His-tagged AtYY1, S284D, and S284A proteins were incubated with plant extract from 10-day-old *Arabidopsis* seedlings for an extended period of time. As shown in **Figure 4C**, S284D was notably more stable than the wild-type AtYY1 protein, whereas S284A was clearly more unstable than both the wild-type AtYY1 and the S284D proteins. This *in vitro* degradation assay supports the notion that S^284^ phosphorylation can enhance the stability of the AtYY1 protein.

### The S^284^ Phosphorylation Site Affects Plant Response to ABA in *Arabidopsis*

Because S^284^ phosphorylation can increase the transcriptional activity and protein stability of AtYY1, it may be effective in regulating the biological function of AtYY1 in ABA signaling (Li et al., 2016). To complete a functional characterization of S^284^ phosphorylation in *Arabidopsis*, we generated three types of complementary transgenic lines by transferring *AtYY1, S284D*, or *S284A* under the control of its own promoter to *yy1* mutants (*pAtYY1::AtYY1, pAtYY1::S284D, pAtYY1::S284A*). Using qRT-PCR, we found no significant difference in the *AtYY1* transcript levels present in the wild-type and the complementary transgenic lines in control conditions (Supplementary Figure S3).

**FIGURE 5:**
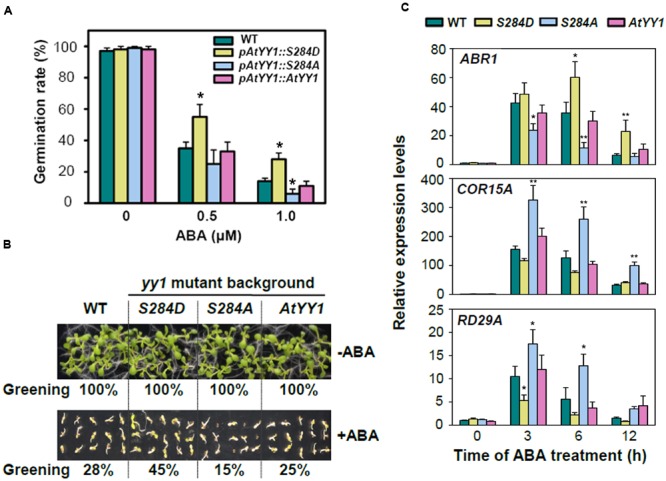
**S^284^ phosphorylation site affects the plant response to ABA in *Arabidopsis*. (A)** The effect of S^284^ phosphorylation on the seed germination rate with or without ABA treatment. *pAtYY1::AtYY1, pAtYY1::S284D*, and *pAtYY1::S284A* transgenic plants were generated on the *yy1* mutant background. The seed germination rate was determined after incubation on MS medium containing 0, 0.5, or 1.0 μM ABA for 2 days. ^∗^*P* < 0.05 (*t*-test). **(B)** The effect of S^284^ phosphorylation on cotyledon greening with or without 0.5 μM ABA treatment for 8 days. The greening percentages of each line are shown at the bottom of each panel. **(C)** Expression analysis of *ABR1, COR15A* and *RD29A* in the wild-type and transgenic plants under 100 μM ABA treatment. ^∗^*P* < 0.05 and ^∗∗^*P* < 0.01 (*t*-test).

The response to ABA was then compared among the wild-type and complementary transgenic lines. After 2 d of germination on MS medium containing 0.5 or 1.0 μM ABA, the seed germination rate of *pAtYY1::S284D* was notably higher than that of the wild-type and other two complementary lines **(Figure 5A**). Similar results were observed in the cotyledon greening assay, in which the *pAtYY1::S284D* transgenic lines showed a higher greening ratio than the other lines at 0.5 μM ABA (**Figure 5B**). In contrast, *pAtYY1::S284A* transgenic lines showed more sensitive phenotype to ABA than the wild-type. As AtYY1 can positively regulate the expression of *ABR1*, and negatively regulate the expression of ABA responsive genes such as *COR15A* and *RD29A* (Li et al., 2016), we next compared the expression of *ABR1, COR15A*, and *RD29A* among the wild-type and complementary transgenic lines. Compared with the wild-type, ABA-mediated induction of *ABR1* was substantially enhanced in the *pAtYY1::S284D* lines, while it was impaired in the *pAtYY1::S284A* lines (**Figure 5C**). For *COR15A* and *RD29A*, their expression levels were both reduced in the *pAtYY1::S284D* lines, whereas increased in the *pAtYY1::S284A* lines compared with the wild-type following ABA treatment (**Figure 5C**). Therefore, our data clearly suggest that the mimicked phosphorylation of AtYY1 S^284^ confers less sensitivity to ABA in *Arabidopsis* plants and thus strengthens the effect of AtYY1 as a negative regulator in the ABA response (Li et al., 2016).

Previous studies have identified abundant kinases and phosphatases implicated in ABA signaling, including SnRK2s, SnRK3s/CIPKs, CDPKs/CPKs, PP2As, and PP2Cs, suggesting that reversible phosphorylation is crucial in modulating ABA signal transduction events (Cutler et al., 2010). In this study, we identified an ABA repressor, AtYY1, as a potential target of CKII, indicating that CKII is also a regulator of ABA signaling. The mimicked phosphorylation of AtYY1 S^284^ by CKII effectively strengthened the function of AtYY1 as a negative regulator in the ABA response. However, CKII α subunit mutants are hyposensitive to the ABA and NaCl-induced blockade of seed germination and cotyledon greening in a synergistic manner (Mulekar et al., 2012; Wang et al., 2014), while the *yy1* mutant is hypersensitive to ABA and salt (Li et al., 2016). This contradiction may be explained by the fact that CKII has abundant potential target substrates in plants and can act as an important regulator in the ABA response by affecting the activity and stability of multiple downstream negative and/or positive regulators. Further investigation will be required to identify these CKII targets in ABA signaling and clarify the authentic physiological effect of AtYY1 S^284^ site by monitoring its phosphorylation state *in vivo*.

## Conclusion

In this study, we demonstrated that AtYY1 can interact with the CKII β subunits CKB3 and CKB4, and be phosphorylated by CKII at S^284^ site. The phosphorylation of AtYY1 S^284^ site by CKII enhances its transcriptional activity and protein stability. The *pAtYY1::S284D* (a hyperphosphorylated form) transgenic plants in the *yy1* mutant background are hyposensitive to ABA, whereas the *pAtYY1::S284A* (a phosphorylation-deficient form) transgenic plants are hypersensitive to ABA compared with the wild-type. Together, these data provide evidence that the CKII-mediated phosphorylation of AtYY1 S^284^ site strengthens the effect of AtYY1 as a repressor in ABA signaling.

## Author Contributions

The authors have made the following declarations about their contributions: X-YW and TL designed research, X-YW and TL performed research, X-YW and TL analyzed data, X-YW and TL drafted the manuscript.

## Conflict of Interest Statement

The authors declare that the research was conducted in the absence of any commercial or financial relationships that could be construed as a potential conflict of interest.

## Footnotes

1 http://prosite.expasy.org/
