# Supplementary material for: A Casein Kinase II Phosphorylation Site in AtYY1 Affects Its Activity, Stability, and Function in the ABA Response
Source: Front Plant Sci. 2017 Mar 13;8:323. doi: 10.3389/fpls.2017.00323 (PMC5346550; doi:10.3389/fpls.2017.00323)

Table S1. Primers used in this study.

**Primers used for qRT-PCR (5′-3′)**	
YY1-F	GAGAACATCCAGGGCATTTA
YY1-R	TGGCAGGCGAAGAGGTAGA
CKB3-F	TCCCTTTTCCTTCACATCTACT
CKB3-R	AACATCTCACTGTTGGAAGCA
CKB4-F	AAGGACAAAGATTCTGTCCCTT
CKB4-R	CAGCAGCTGATTCCACCATC
ABR1-F	GAAGGTAGCGGAGAAGGGA
ABR1-R	CGAGGTTGGTCCCCACTAA
COR15A-F	CTTTCCACAGCGGAGCCA
COR15A-R	CCAATGTATCTGCGGTTTCA
RD29A-F	TCAACACACACCAGCAGCAC
RD29A-R	ATCGGAAGACACGACAGGAA
Actin-F	TTGACTACGAGCAGGAGATGG
Actin-R	CAAACGAGGGCTGGAACAAG
**Primers used for vector construction (5′-3′)**	
YY1-Bifc-F	CGGGATCCATGGATCATCAAAATTATC
YY1-Bifc-R	ACGCCTCGAGATCTTCATACTCGGTCTCT
CKB3-Bifc-F	CGGGATCCATGTACAAGGAACGTAGTGGA
CKB3-Bifc-R	ACGCCTCGAGTGGTTTGTGTACCTTGAAG
CKB4-Bifc-F	CGGGATCCATGTACAAGGATCGGAGTGGA
CKB4-Bifc-R	ACGCCTCGAGTTGTTTGTTGTGTACCTTAAA
YY1-3301-F	CGGGATCCATGGATCATCAAAATTATC
YY1-3301-R	ACGCACGTGCTAATCTTCATACTCGGTC
YY1-P-F	GGGAGCTCTGGGTCAGTGAAGACAATAATGC
YY1-P-R	CGGGATCCTGATTTATTTGGGTAAAGGTGGTT

Figure S1. qRT-PCR analysis of *CKB3* (A) and *CKB4* (B) expression under ABA treatment. 10-day-old seedlings were treated with 100 μM ABA for the indicated times (0, 3, 6, or 12 h).

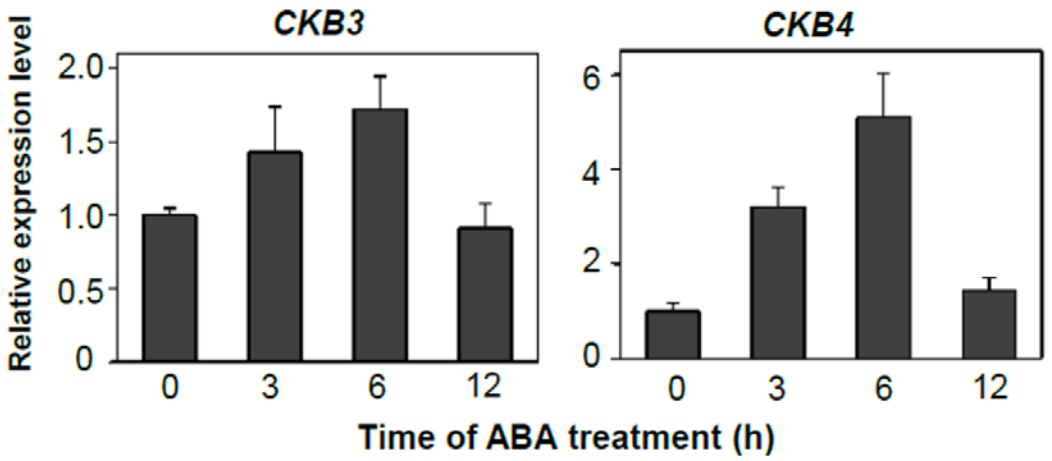

Figure S2. Five phosphorylated AtYY1 peptides were identified using LC-MS/MS after an *in vitro* phosphorylation assay with CKII. The amino acids labeled with a P indicate that the sites are phosphorylated. The fragment ions assigned to both y-ions and b-ions are labeled.

A

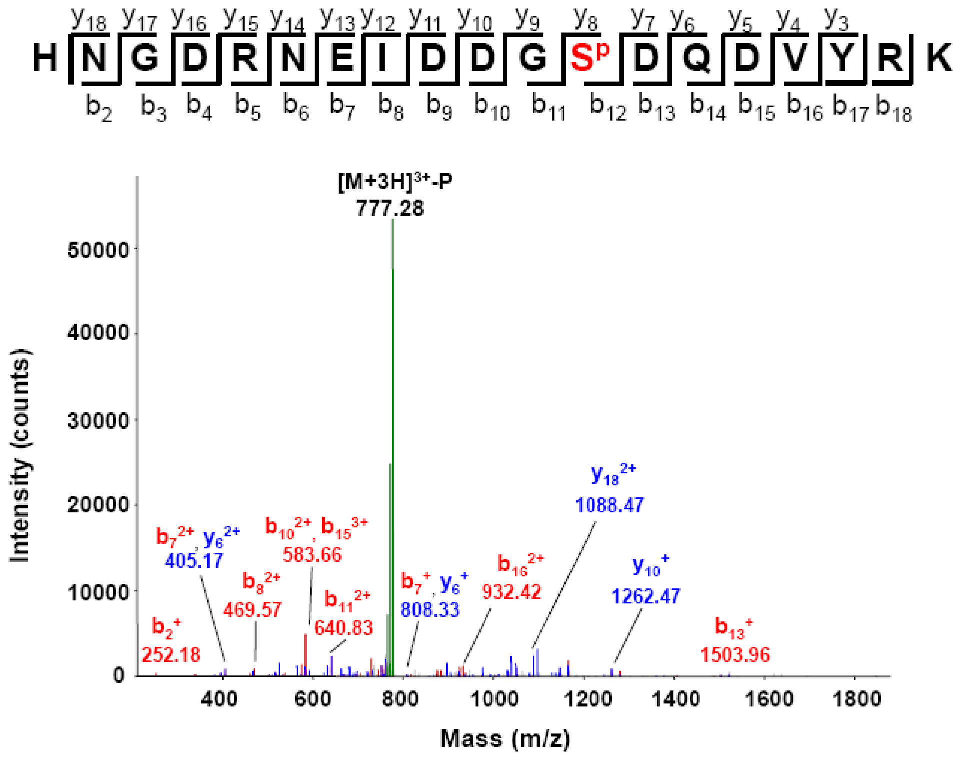

B

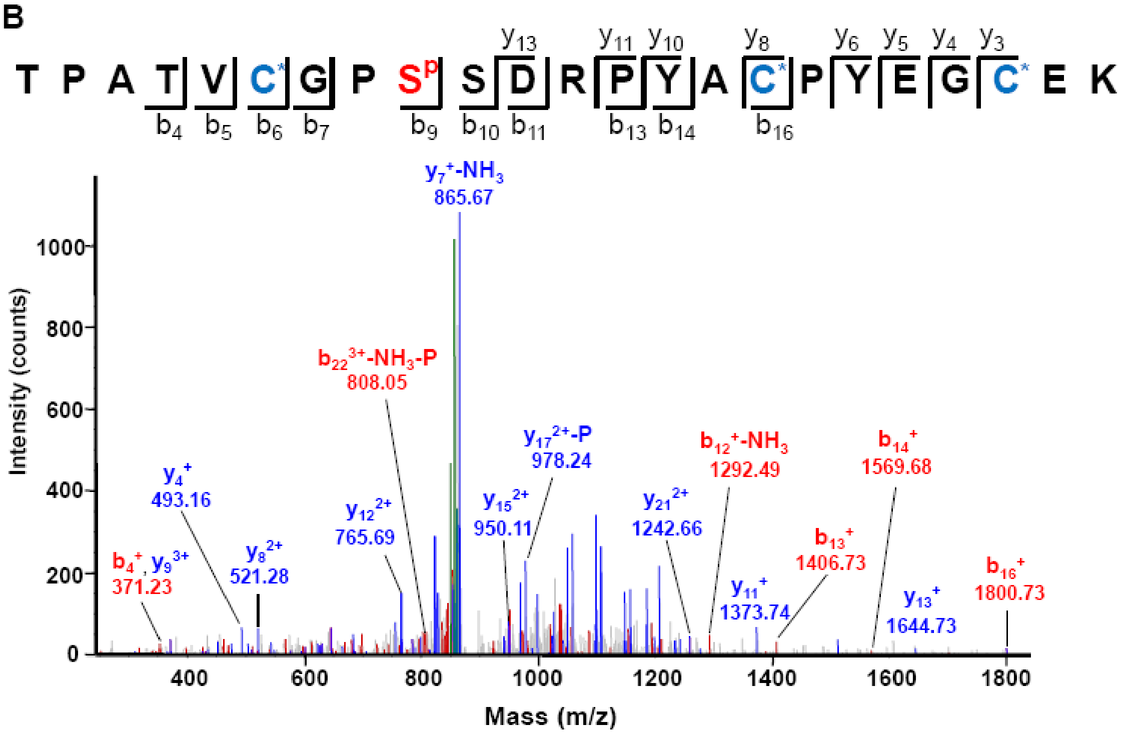

T P A T V C* G P Sp S D R P Y A C* P Y E G C* E K
y13 y11 y10 y8 y6 y5 y4 y3
b4 b5 b6 b7 b9 b10 b11 b13 b14 b16
y7+-NH3 865.67
b22 3+-NH3-P 808.05
y17 2+-P 978.24
b12+-NH3 1292.49
b14+ 1569.68
y4+ 493.16
y12 2+ 765.69
y15 2+ 950.11
y21 2+ 1242.66
b13+ 1406.73
b16+ 1800.73
b4+, y9 3+ 371.23
y8 2+ 521.28
y11+ 1373.74
y13+ 1644.73
Intensity (counts)
Mass (m/z)

C

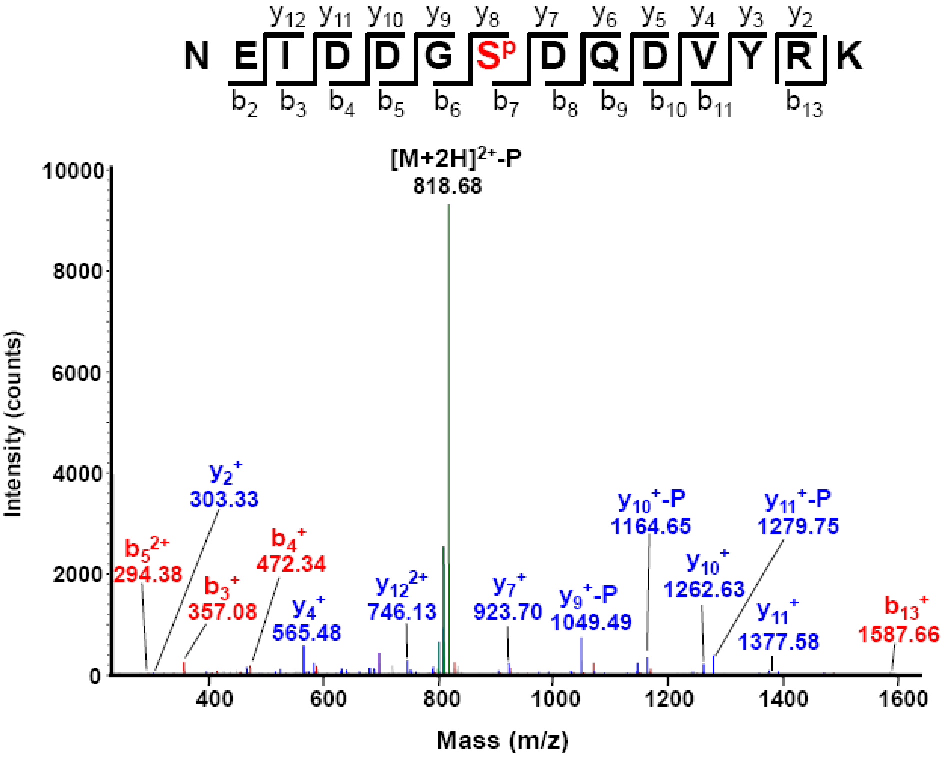

N E I D D G Sp D Q D V Y R K
y12 y11 y10 y9 y8 y7 y6 y5 y4 y3 y2
b2 b3 b4 b5 b6 b7 b8 b9 b10 b11 b13
[M+2H]2+-P 818.68
y2+ 303.33
b5 2+ 294.38
b4+ 472.34
b3+ 357.08
y4+ 565.48
y12 2+ 746.13
y7+ 923.70
y9+-P 1049.49
y10+-P 1164.65
y10+ 1262.63
y11+-P 1279.75
y11+ 1377.58
b13+ 1587.66
Intensity (counts)
Mass (m/z)

D

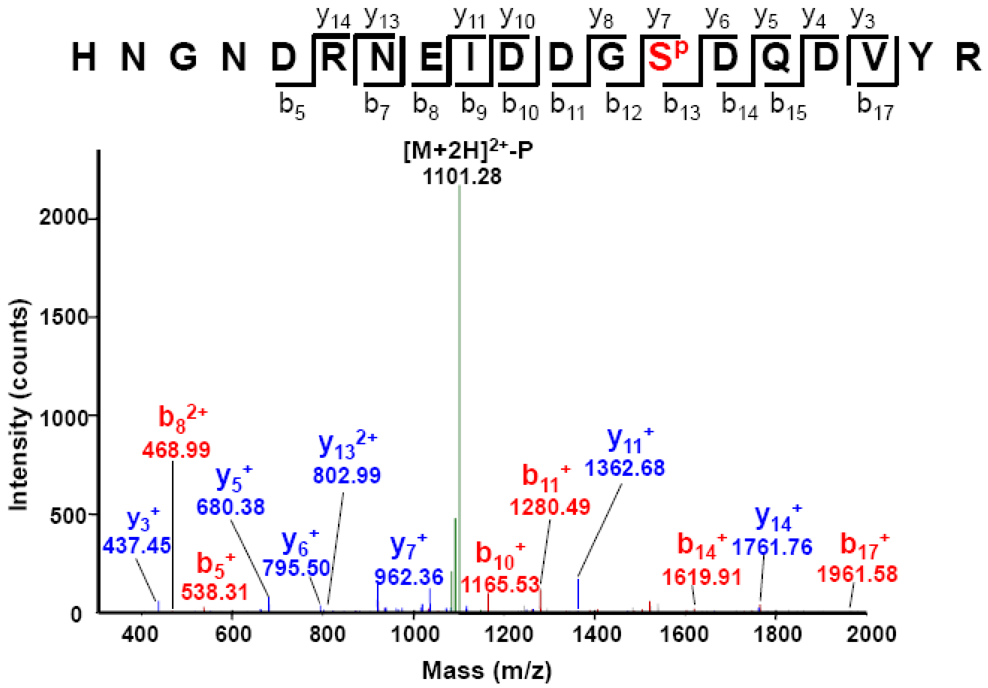

H N G N D R N E I D D G S^p D Q D V Y R
y14 y13 y11 y10 y8 y7 y6 y5 y4 y3
b5 b7 b8 b9 b10 b11 b12 b13 b14 b15 b17
[M+2H]2+-P
1101.28
Intensity (counts)
Mass (m/z)
b8 2+ 468.99
y3 + 437.45
y5 + 680.38
b5 + 538.31
y13 2+ 802.99
y6 + 795.50
y7 + 962.36
b10 + 1165.53
b11 + 1280.49
y11 + 1362.68
b14 + 1619.91
y14 + 1761.76
b17 + 1961.58

E

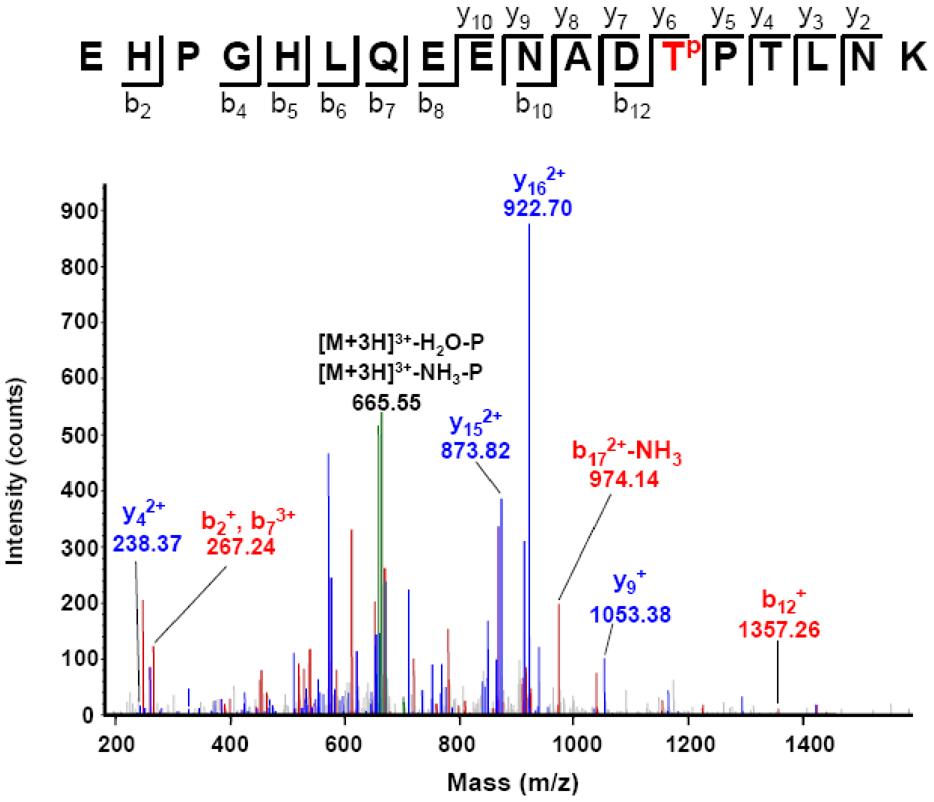

E H P G H L Q E E N A D T^p P T L N K
y10 y9 y8 y7 y6 y5 y4 y3 y2
b2 b4 b5 b6 b7 b8 b10 b12
y16 2+ 922.70
[M+3H]3+-H2O-P
[M+3H]3+-NH3-P
665.55
y15 2+ 873.82
b17 2+-NH3 974.14
y4 2+ 238.37
b2 +, b7 3+ 267.24
y9 + 1053.38
b12 + 1357.26
Intensity (counts)
Mass (m/z)

Figure S3. qRT-PCR analysis of *AtYY1* expression in the seedlings of WT, *pAtYY1::S284D*, *pAtYY1::S284D* and *pAtYY1::AtYY1* plants in control conditions.

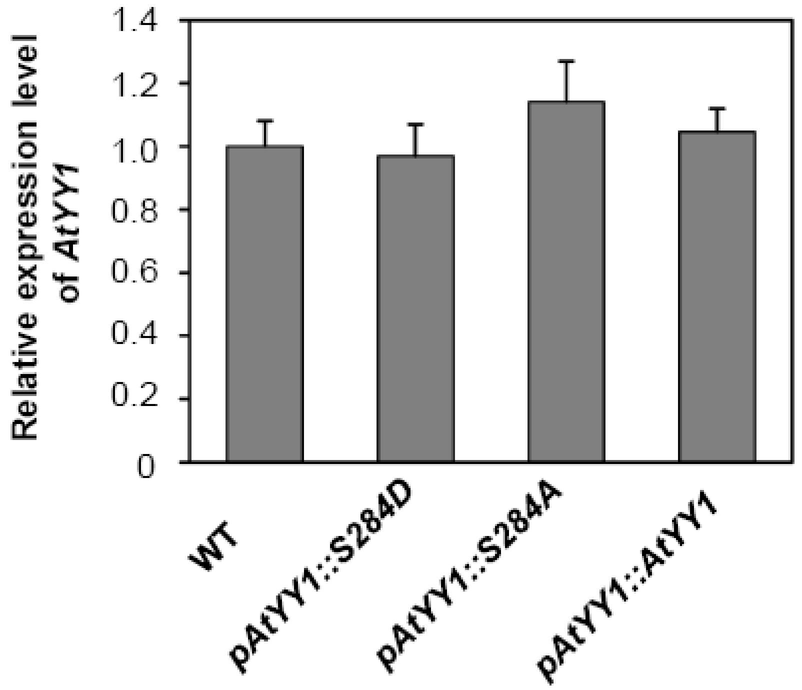

Supplement: Supplementary file 1 [file Data_Sheet_1.pdf]
